# Identifying Causal Variants Associated with Brain Atrophy Using Cross‐sectional and Longitudinal MR Imaging and Whole Genome Sequencing in Koreans

**DOI:** 10.1002/alz70856_104107

**Published:** 2025-12-25

**Authors:** Yoontae Kim, Lindsay A. Farrer, Kun Ho Lee, Jungsoo Gim

**Affiliations:** ^1^ BK21 FOUR, Department of Integrative Biological Sciences, Chosun University, Gwangju, Jeon‐La, Korea, Republic of (South); ^2^ Boston University Chobanian & Avedisian School of Medicine, Boston, MA, USA; ^3^ Gwangju Alzheimer's and Related Dementia (GARD) Cohort Research Center, Chosun University, Gwangju, Korea, Republic of (South); ^4^ BK21 FOUR, Department of Integrative Biological Sciences, Chosun University, Gwangju, Korea, Republic of (South); ^5^ Korea Brain Research Institute, Daegu, Korea, Republic of (South); ^6^ Chosun University, Gwangju, Jeon‐La, Korea, Republic of (South); ^7^ Well‐ageing Medicare Institute, Chosun University, Gwangju, Korea, Republic of (South); ^8^ Chosun University, Gwangju, Korea, Republic of (South)

## Abstract

**Background:**

Brain atrophy is a hallmark of many neurological disorders and is strongly associated with cognitive decline. Recent advancements in neuroimaging, particularly MRI, have facilitated the detection of structural changes in the brain. These changes may be influenced by genetic variations, including rare variants often overlooked in traditional genome‐wide association studies (GWAS). Understanding the genetic factors associated with brain atrophy can provide crucial insights into its mechanisms and support the development of novel diagnostic and therapeutic strategies. However, most studies have been conducted on predominantly Caucasian, with limited research on other ethnic groups.

**Method:**

We conducted a cross‐sectional and longitudinal imaging genetics analysis using 9,841 structural MRIs from 5,870 Korean subjects and whole‐genome sequencing data from the Gwangju Alzheimer's & Related Dementia (GARD) cohort, supplemented with imputed microarray data from the Korean reference panel. Cross‐sectional and longitudinal GWAS were performed using 16 subcortical volumes and 62 cortical thickness measurements to identify genetic variants associated with structural brain changes.

**Result:**

Our analysis identified 43 genome‐wide significant loci associated with brain atrophy based on adjusted *p*‐values (*p* < 6.4110^‐10^). Post‐GWAS analyses, including fine mapping, functional annotation, and pathway enrichment, provided further insights into the biological relevance of these loci. These findings highlight the role of genetic factors in driving region‐specific brain changes.

**Conclusion:**

This study provides new insights into the genetic underpinnings of brain atrophy, emphasizing the influence of genetic variations on structural brain changes. By identifying significant loci associated with brain atrophy, these findings contribute to a deeper understanding of neurodegenerative processes. Future research may leverage these results to develop early diagnostic tools and targeted therapies for neurological disorders characterized by brain atrophy.

